# A call for change from impersonal risk assessment to a relational approach: professionals’ reflections on the national guidelines for suicide prevention in mental health care in Norway

**DOI:** 10.1080/17482631.2020.1868737

**Published:** 2021-01-06

**Authors:** Kristin Espeland, Heidi Hjelmeland, Birthe Loa Knizek

**Affiliations:** Department of Mental Health, Faculty of Medicine and Health Sciences, NTNU, Trondheim, Norway

**Keywords:** Suicide prevention, risk assessment, mental healthcare, qualitative research, relational approach

## Abstract

**Purpose:**

The purpose of the study is to explore how professionals working with suicide prevention experience the influence of the national guidelines on mental healthcare, and to gather recommendations for which steps to take next.

**Methods:**

This is a qualitative study with an explorative design. We interviewed 22 professionals responsible for implementing suicide prevention action plans and guidelines, and/or conducting relevant research. We analysed the data by means of thematic analysis.

**Results:**

We found that the participants had an ambivalent view on risk assessment—it may be a tool, but it may also compromise other important aspects in prevention. Moreover, the possibility of liability has resulted in the need for self-protection. Instead, the participants recommended a relational approach to suicide prevention.

**Conclusions:**

We found that the emphasis on standardized suicide risk assessment has negatively influenced suicide prevention in mental healthcare, and an approach emphasizing relational aspects is recommended. However, the prevailing objectifying concept of knowledge, the epistemological debate and the emergence of the New Public Management ideology may obstruct a fundamental emphasis on relationships. A paradigm shift in mental healthcare is called for with respect to the concept of knowledge, which forms our understandings and practices.

## Introduction

In the Western world, suicide prevention is mostly the responsibility of the mental healthcare services (Marsh, 2010; Walby et al., 2018), and the World Health Organization (WHO, 2014) has pointed out the importance of having access to healthcare and improving the quality of care in suicide prevention. In 2008, the Norwegian Directorate of Health and Social Affairs published the *National Guidelines for Prevention of Suicide in Mental Health Care* hereafter referred to as “the national guidelines” (Norwegian Directorate of Health and Social Affairs, 2008). These guidelines were published six years after the Norwegian hospital reform in 2002, which was influenced by the New Public Management (NPM) ideologies that emerged in the 1970s–1990s (Jespersen & Wrede, 2009; Lægreid et al., 2005). NPM arose out of the growing critique of increasing expenditures, inefficiency and lack of service orientation, as well as criticism of the medical professions’ monopoly in specialist healthcare (Jespersen & Wrede, 2009). With its roots in the market economy and private sector management, the NPM reform of the public sector aimed to improve efficiency, productivity, quality and accountability (Blomgren & Sahlin, 2017; Lægreid et al., 2005). Within healthcare, the purpose of the NPM reform was to improve cost control, distribute health resources more equally, reduce the size of government and limit the medical professions’ control in the governance of healthcare. Thus, the aim was to develop a more decentralized system and to empower patients (Byrkjeflot, 2005, 2011). For example, the NPM reform intensified monitoring and reporting requirements (Lægreid et al., 2005; Wyller et al., 2013), where guidelines were one of the techniques applied (Blomgren & Sahlin, 2017).

The national guidelines aimed to improve the mental health services through standardized and quality assured treatment and to raise the competence of mental healthcare workers. To accomplish these goals, the guidelines comprise recommendations within seven areas. In this article, we focus on the area referring to identification and assessment of suicide risk. In addition to the recommendations, the guidelines include appendices with suggestions for procedures relating to risk-factor-based suicide risk assessment as well as training in identification and assessment of suicide risk. Whilst it is claimed that the national guidelines are not to be binding as such, they are still aimed at governing the choices made: “Professionals will contribute to fulfilling the requirements of justifiability by following the guidelines” (Norwegian Directorate of Health and Social Affairs, 2008, p. 10). It is also mandatory to report all suicides and serious suicide attempts to the Norwegian Board of Health Supervision which then evaluates whether therapists have conducted risk assessments according to procedure (Norwegian Directorate of Health and Social Affairs, 2008). Since the guidelines see deviation from the procedure as a violation of both the Health Personnel Act and the Specialized Health Services Act, they are, in practice, binding (Granlund, 2014).

The guidelines were implemented through regional courses led by representatives from the National Centre for Suicide Research and Prevention, as well as the five regional resource centres on violence, traumatic stress and suicide prevention. The aim of the courses was to prepare the participants (mostly specialists in medicine and psychology) to arrange guideline courses in their respective regions (Ness, 2009). Following the implementation of the national guidelines, the chapter and appendices relating to suicide risk assessment received much attention in the media. In the daily press as well as in professional journals, clinicians and researchers debated numerous problems with the guidelines. In professional journals, the emphasis on risk assessment was discussed. Some maintained that suicide risk assessment could impede good treatment and make therapists less aware of the patient`s needs (Hagen et al., 2014; Stangeland et al., 2018) and that the guidelines contributed to unattainable expectations that professionals can prevent suicide if they conduct risk assessment according to procedure (Hagen et al., 2014). Others claimed that risk assessment would increase the safety and quality for all patients (Mehlum et al., 2014) and that risk assessment is useful in a short-term perspective (Ekeberg & Hem, 2017).

The purpose of suicide risk assessment is to categorize patients as either at a “high”, “medium” or “low” risk of suicide (Large & Ryan, 2014; Murray, 2016). Some assume that there are valid markers of increased risk of suicide, and when these markers are identified, interventions can improve the management of suicide risk (Chu et al., 2015). However, because of low predictive value (Fosse et al., 2017; Large et al., 2018; Murray, 2016) and that overreliance on identifying risk factors may provide false reassurance and potentially be dangerous (Chan et al., 2016), the utility of suicide risk assessment has been questioned. Already in 1983, Pokorny (1983) found in a prospective study that 93.6% of patients categorized as “high risk” were false positives. Moreover, more than half of the suicides had been categorized as “low risk”; hence, they were false negatives. Therefore, some researchers recommend that it is time to abandon risk-factor-based suicide risk assessment in favour of alternative approaches (Large & Ryan, 2014; Large et al., 2011; Murray, 2016). For example, White et al. (2016) envisage a future with relational, strengths-based, culturally responsive and social justice-oriented approaches. The importance of relational aspects and approaches to suicide prevention in mental healthcare has been emphasized in several qualitative studies (Østlie et al., 2018; Rasmussen & Dieserud, 2018; Talseth et al., 2003; Vatne & Nåden, 2016, 2018). Emphasizing the therapeutic alliance, “the active and purposeful collaboration between patient and therapist” (Michel, 2011, p. 14), is one example of a relational approach to suicide prevention.

A revision of the national guidelines has been advised (Walby, 2018), mostly due to the criticism of the emphasis on standardized risk assessment. By interviewing professionals implementing the national guidelines (and/or involved in relevant research), the purpose of this study is to explore how professionals experience the influence of the national guidelines on mental healthcare, and to gather recommendations for which steps to take next. The knowledge, experiences and recommendations of these professionals may contribute to improving suicide prevention in mental healthcare.

## Method

This is an explorative qualitative study based on semi-structured interviews that have been analysed by means of thematic analysis (Braun & Clarke, 2006).

### Participants

The participants were 22 professionals, eight men and 14 women with a median age of 55.5 years, working with implementation of national guidelines and action plans, and/or relevant research throughout Norway. Some of the participants currently worked with or had previously worked with suicidal patients. Most of the participants were educated in the field of mental healthcare (psychiatrists, psychologists, mental health nurses) and some within in the social sciences. Because of their experience with implementation work and/or relevant research, many of the participants were in contact with therapists and other clinical professionals working with suicidal patients. Thus, the participants are able to reflect on and describe how they experience the influence of the national guidelines and what they recommend to do next.

The first author emailed the leaders of the five regional resource centres on violence, traumatic stress and suicide prevention, the National Centre for Suicide Research and Prevention (NSSF), the Norwegian Institute of Public Health (FHI), and the relevant department at Oslo University Hospital with a request to invite relevant employees to participate. With the leaders’ consent, the first author sent an email and invited relevant employees to participate. In addition, some former employees with relevant experience for this study were invited directly. Employees from all the approached institutions accepted our invitation, with the exception of NSSF, which unfortunately chose not to participate. This means we have participants from all the other institutions. The participants’ experience of work in suicide prevention and/or relevant research ranged from nine to 35 years. Eighteen of the 22 participants had worked with suicide prevention in one way or another for more than 10 years.

### Data collection

The first author conducted all the interviews, with the exception of two that the third author conducted because the first author was acquainted with these two participants. All participants except one who was interviewed at home were interviewed at their respective workplaces. The interviews, held in 2017, lasted from 32 to 120 minutes (average about 90 minutes). A semi-structured interview guide was used, where the main themes were the participants’ experiences of 1) working with national action plans and strategies, their views on 2) the suicide rate, 3) prevention in the future, and 4) user involvement in suicide prevention. In this article, we focus on the first three themes, which are relevant to explore the purpose of the study. The participants were encouraged to share both positive and negative experiences of the suicide prevention work in Norway. Where appropriate, the interviewer asked follow-up questions, such as “could you please elaborate”, “what do you mean by that”, and “have I understood you correctly in that … ” in order to clarify the participants’ statements and experiences (Brinkmann & Kvale, 2015). All the interviews were recorded and transcribed verbatim.

### Analysis

We used thematic analysis as outlined by Braun and Clarke (2006) to analyse the data. Thematic analysis comprises six phases where the aim is to notice and look for patterns, meanings and issues, and to report experiences and the participants’ actual situation (Braun & Clarke, 2006). Initially, the first and second author familiarized themselves with the data, the second author read anonymized transcripts, by reading the transcripts and noting down what each participant said concerning the guidelines. Guided by the purpose of the study, the first author moved on to the second and third phase, coding interesting features and developing preliminary themes based on codes belonging together. The first author discussed the preliminary themes with the other authors, and then moved on to check the themes against coded data extractions. As described by Braun and Clarke (2006), the analysis is a *recursive* process with movement back and forth between the phases, and thus we moved back and forth between the data set, the analysis and the written text to develop the final three themes (see Table I). The writing thus became an integral part of the analysis. The analysis is inductive and the themes are identified at a semantic/explicit level (Braun & Clarke, 2006), not looking beyond what the participants said and not engaging with literature until the last phase of writing.

**Table I.** Example of the analysis process leading to the theme “An ambivalent view on risk assessment”

Step 1: Example of one interesting feature in the transcript	*I think that they [the guidelines] are very, very focused on risk assessments. And I believe maybe they are, that it’s an important part, but that it is at the cost of so many other things. It’s like that becomes the focus, and I think that the huge focus on these risk assessments means that perhaps you lose sight of the big picture and other aspects that perhaps are just as important when working with suicide prevention in specialist mental healthcare as well*
Step 2: Codes from the feature	Focused on risk assessment; important with focus on risk assessment; risk assessment at the sacrifice of other important aspects; other aspects are just as important
Step 3: Developing preliminary themes based on codes belonging together (codes retrieved from several interesting features)	Necessary to put suicide prevention on the agenda (codes: important to address suicidality; need some kind of recipe; risk assessment is made visible; important with focus on risk assessment; guiding the direction) Unsuccessful emphasis on risk assessment (codes: the emphasis is unfortunate; the Board of Health Supervision emphasis reinforced emphasis on risk assessment; consequences for both therapists and patients; risk assessment at the sacrifice of other important aspects; other aspects are just as important)
Step 4: Review theme	Moving back to the data set, we found that when the participants talked about how the guidelines contributed to put suicide prevention on the agenda and the benefits with suicide risk assessment, they also talked about the disadvantages with a narrow emphasis on risk assessment. Consequently, the preliminary themes “Necessary to put suicide prevention on the agenda” and “Unsuccessful emphasis of risk assessment” seemed to be interdependent.
Step 5: Define name and theme	Description: The participants described both benefits and disadvantages with risk assessment. The final theme *An ambivalent view on risk assessment* captured some of the complexity regarding the discussion about risk assessment.

### Ethical considerations

The second author led one of the regional centres from its establishment in 1997 through 2003 but since then has not had any formal role in suicide prevention work. The first and third authors have never been involved in suicide prevention work in Norway. None of the authors have been involved in the development or implementation of the national guidelines. The Norwegian Centre for Research Data approved the study (reference 53298/3/SMS). The participants signed an informed consent form and were informed about the option to withdraw from the study (up until the time of publication) without needing to provide a reason. We treated the data confidentially and do not report the participants’ specific educational background or workplace to ensure their anonymity. For the same reason, all participants are referred to as “she”.

## Findings and discussion

Although suicidality is well known in the mental healthcare field, several participants stated that the national guidelines were important because they draw attention to the topic and put suicide prevention on the agenda more systematically. As in the public debate, the chapter and appendices on risk assessment received the most attention in this study. Thus, the first two themes elaborate on the participants’ reflections on and experiences of the emphasis on risk assessment and its consequences, outlined as “an ambivalent view on suicide risk assessment” and “self-protection”. The last theme, “a relational approach to suicide prevention”, elaborates on the participants’ recommendations for suicide prevention in mental healthcare.

### An ambivalent view on suicide risk assessment

Only one of the seven chapters in the national guidelines focuses on standardized risk-factor-based suicide risk assessment (Norwegian Directorate of Health and Social Affairs, 2008). However, some participants claimed that directives from the national centre resulted in a strong and constraining emphasis on this type of risk assessment when the guidelines were implemented, which they found problematic. Moreover, most of the participants described an ambivalent view on risk assessment—it may be a useful tool for structuring the encounter with suicidal patients, but it may also compromise other important aspects in suicide prevention. The following excerpt illustrates how risk assessment may be a tool:

*But I think that having a framework for how we should carry out an assessment is important, maybe especially also for new ones [new employees], because what are we supposed to ask about, what is it that we should do, how should we relate to this … (P17)*

From this excerpt, it appears as if having some type of structure in the encounter with suicidal patients is important, and that risk assessment thus may be a useful tool for guiding what to ask about, what to do and how to relate to suicidality. Another participant believed risk assessment could be useful when initiating safety measures and treatment. Moreover, one participant thought that a systematic focus was important to ensure that patients had the opportunity to reveal suicidal thoughts. Hence, from the participants` accounts, it seems like the chapter and attachment on suicide risk assessment may contribute a framework for talking and asking about suicidality.

On the other hand, most of the participants also described a too narrow emphasis on standardized risk assessment, for instance:

*I think that they [the guidelines] are very, very focused on risk assessments. And I believe maybe they are, that it’s an important part, but that it is at the cost of so many other things. It’s like that becomes the focus, and I think that the huge focus on these risk assessments means that perhaps you lose sight of the big picture and other aspects that perhaps are just as important when working with suicide prevention in specialist mental healthcare as well. (P2)*

In our interpretation, it seems like a constraining emphasis on risk assessment may compromise other important aspects of suicide prevention. This participant believed that a focus on risk assessment made professionals less attentive to the “big picture” and that other aspects are just as important in the prevention of suicide. In accordance with the excerpt above, other participants saw risk assessment as a simplification of the real and complex situations, thus disregarding the context of the suicidality. One participant felt that the relationship with the patient and the treatment aspect were not given sufficient attention due to the emphasis on risk assessment. Another participant feared that risk assessment *per se* was being treated as more important than safety and treatment. She stated that risk assessment has been highlighted as the only solution to suicide prevention, which she believed has been a total failure. Hence, from the participants` accounts, it seems that risk assessment may be a tool to operationalize and facilitate conversations about suicidality between patients and therapists, but that a strong and constraining emphasis may be detrimental when considering the complexity of suicidality, relational aspects and treatment.

The limitations of risk-factor-based suicide risk assessment that the participants have described above have been acknowledged and discussed by several researchers (Chan et al., 2016; Large et al., 2018), where, for example, Chan et al. (2016) claim that “the idea of risk assessment as risk prediction is a fallacy and should be recognized as such” (p. 282). However, some researchers claim that risk assessment is still important but that a therapeutic risk assessment should be the norm (Wortzel et al., 2017). For instance, risk assessment is a component in the suicide-specific therapeutic framework *Collaborative Assessment and Management of Suicidality* (CAMS; Jobes, 2011). In CAMS, the patient and the therapist make the assessment in collaboration, and it is “incumbent on the clinicians to endeavor to understand the suicidal experience through the eyes of their patient” (Jobes, 2011, p. 215). Thus, the emphasis is not on risk assessment *per se*, but on the collaborative relationship between patient and therapist. This approach seems to be more in accordance with the participants’ request—while there is a need for a framework for talking about suicidality, it should not come at the cost of relational aspects.

### Self-protection

Working with suicidal patients is known to be an emotional endeavour (Hagen, Knizek et al., 2017). From the participants’ accounts, it seems that the constraining emphasis on standardized risk assessment has had some unfortunate side effects and added to the emotional burden of caring for suicidal patients, consequently supporting the need for therapists to protect themselves. The participants relate this to how the Norwegian Board of Health Supervision followed up the guidelines after their implementation:

*So these guidelines had such a status that they had an impact, and the Board of Health Supervision immediately showed that they were interested in whether or not they were being complied with. Now it has been like, what they [Board of Health Supervision] have been interested in, has been narrow. So much of what has happened afterwards has been about the use of risk assessments. (P7)*

This statement indicates that the national guidelines had a major impact and the participant found the Norwegian Board of Health Supervision’s restricted focus on risk assessment problematic. The Board investigates cases where suicides/suicide attempts have occurred to ascertain whether or not the therapists have conducted a risk assessment in accordance with the guidelines. If such an assessment has not been conducted, this is regarded as deviation from procedure (Norwegian Directorate of Health and Social Affairs, 2008). The aim is to improve practice but many of the participants believe this has had a negative impact on therapists’ clinical work:

*And the Board of Health Supervision that measures, that uses the guidelines in their supervisions, insists that you have to comply, you have to assess suicide risk, if a patient takes his/her life and suicide risk assessment was not conducted such and such a long time before the suicide happened, then it is a deviation. So I think that the guidelines in many cases have been more for the system than for the patient, and then the result is that if therapists have followed them [risk assessment] then they have covered themselves because they have done the risk assessment at the right times. So I think that has been, yes, very, very unfortunate. (P5)*

According to this statement, therapists are obliged to conduct risk assessment in accordance with the guidelines in order to meet requirements and avoid liability in the event a patient takes his/her own life. Other participants also used the phrase “cover yourself” when they talked about the consequences of the emphasis on risk assessment. It seems that several participants believe therapists might practice risk assessment as insurance in case a patient takes his/her life. If therapists conduct risk assessments according to procedure, they are “covered” and cannot be blamed for the suicide. Thus, it seems like risk assessment may be more a tool for self-protection than a tool benefiting the patients. Several participants described this emphasis as very unfortunate.

Power (2004) has defined this phenomenon, professionals tied up by system requirements (e.g., risk assessment) to avoid blame, as “secondary risk”. Secondary risk means that experts (e.g., therapists working with suicidality) are held accountable for what they do to such a degree that they become more preoccupied with managing their own risks. Consequently, managing their own risk is “becoming as significant as the primary risks for which experts have knowledge and training” (Power, 2004, p. 14).

In this study, some participant were concerned that the risk assessment process, with a preoccupation with secondary risk, had been adhered to more for the benefit of the system and consequently at the cost of their patients’ care and safety.

*But I think that a field like suicide then, suffers a bit by this, because here there’s a risk that someone could be, could be blamed, that someone is to blame, that someone has made a mistake. And that may be true, they have made a mistake. They should have asked about something or seen something, or discovered something, or whatever, right? And that issue, that’s embedded in the suicide issue, makes you think a lot about your safety, unavoidably. Yes. There, we don’t want the suicides, and we don’t want the blame for them. (P22)*

This statement shows that working with suicide involves a diversity of risks, the risk of doing something wrong (e.g., failing to see signs of suicidality), the risk of being blamed and the risk of patient suicide. When the participant says, “We don’t want the suicides, and we don’t want the blame”, this may reflect a view that professionals working with suicidal patients are caught between the aim of preventing suicide, and the possibility of being blamed for a patient’s suicide. Consequently, therapists need to protect themselves. From most of the participants’ accounts, we get the impression that the possibility of liability and the need to protect themselves has become a burden on therapists. This burden detrimentally affects their encounter with the suicidal patient and their clinical autonomy.

Our findings are in keeping with Undrill (2007) who argues that the management of secondary risk may change how professionals approach patients’ risk and thus may be counter-therapeutic. For example, a study by Felton et al. (2018) indicates that the notion of risk can lead to professionals constructing service users as “objects of risk”. Hagen, Hjelmeland et al. (2017) found “that a high emphasis on instrumental aspects in clinical procedures and interventions might put the care of suicidal patients under pressure” (p. 104).

The findings in our study indicate that an emphasis on system requirements and thus secondary risk may lead to therapists need for self-protection which affects their clinical autonomy and thus makes it difficult to practise what these professionals consider to be good suicide prevention work.

### A relational approach to suicide prevention

In contrast to the current emphasis on standardization and risk-factor-based suicide risk assessment in the national guidelines, the participants in this study called for an emphasis on relational aspects in suicide prevention in mental healthcare:

*I am very opposed to those screening measurements [risk assessments] now, that are introduced everywhere. It is completely hopeless (…) And it becomes so impersonal because I believe in the relation between people if one should manage to prevent suicide. (P11)*

This excerpt reflects the participant’s view against screening measures (risk assessments), even going as far as to call them hopeless and impersonal. What she rather wants as a way of preventing suicide is to put emphasis on the relationship between people. Several other participants used the word “relation” frequently when they talked about recommendations for suicide prevention in mental healthcare (and in general). Thus, we understand that the aspect of relations are important, and that participants are calling for a change of emphasis in suicide prevention; from an impersonal approach (risk assessment) to a relational approach.

The importance of relational aspects is emphasized in the literature as well. According to Deegan (1996), building a relationship is the most powerful tool when working with people. Michel (2011) emphasizes the importance of a therapeutic alliance, which is a collaborative relationship between patient and therapist, and a process where the patient allows the therapist to enter his or her personal world (Michel, 2016). A collaborative relationship between therapist and patient has been found to be a consistent predictor of therapy outcomes (Baldwin et al., 2007; Del Re et al., 2012).

Another participant described the importance of relationships as follows:

*… to be able to establish a relationship that makes it possible for the person concerned to start talking about what is difficult and have trust in that: yes, here I can say what I have to say, here I can share, here I can talk about it. I think, that must be above all what is important (…) But that, the main focus should be on the relation – how to create confidence, what it’s about, and that this must be, yes, that this is what is preventive, and for suicidality, I think. (P12)*

This participant emphasizes how crucial it is to establish a relationship with the suicidal patient, and that this should be the focus of attention. Moreover, the statement indicates that the relationship is a prerequisite for the patient to feel safe enough to share his or her thoughts and feelings. Furthermore, this excerpt suggests that the relationship itself might be preventive in terms of suicidality.

Our findings are in keeping with Vatne and Nåden (2018) who found that a relational approach is crucial because suicidal patients need to be understood regarding the meaning(s) of their wish to die. Therefore, professionals must invite patients to talk about suicidality and make room for experiences of connectedness in the dialogue (Vatne & Nåden, 2016). Vatne and Nåden (2016) found that when suicidal patients experienced connectedness, they became more aware of responsibility in life, and this seemed to have a preventive value. Dialogues and engagement in patients’ suffering can also increase the hope to go on living (Cutcliffe & Barker, 2002; Vatne & Nåden, 2018). In keeping with the literature, several participants in our study believed it was vital for therapists to go down into the “black hole” with the suicidal patient. One participant described it like this:

*So that health personnel dare to be present and be down into the black hole, is extremely important. That you’re not too quick in suggesting that ‘I can help you with this, and you have no reason to, nah you don’t need to die, you, you have so much to live for’. That’s a bit too easy. Working with these people requires that you manage to keep calm and be in what is difficult, that’s what’s helpful. (P3)*

This exerpt reflects a view that an ability to listen to reflections on death and everything that is difficult without resorting to trivializations or simple persuasions is what is needed. In this “black hole”, patients can explore their own situation together with the therapist. However, it seems like this is perceived to be a challenging endeavour. Another participant described “going into the black hole” as frightening and uncomfortable because helpers want to fix the problem. However, several participants pointed out that it was necessary to dare to be present in spite of such feelings.

Our findings are is in keeping with Michel (2016) who asserts that “the therapist’s ability to show empathy for the patient’s suicidal wish and to refrain from trying to talk the patient out of it” (p. 351) is one of the most difficult aspects of the therapeutic alliance. Østlie et al. (2018) highlight the ability to really *listen* to suicidal patients. They found that therapists must engage in a deep listening perspective to expand their understanding of the patient’s private theory of suicidality and their beliefs about a possible cure, and they maintain that this is particularly important for therapists who use standardized methods.

However, the findings in our study suggest that emphasis on risk assessment and concerns about liability may make therapists worried instead of empowering them to go down into the “black hole” and listen to patients. One participant stated:

*… then that anxiety you might have in relation to suicidality, there was, it was turned towards that … if only you had followed the procedure and counted this and that, then no one can get you. And that’s a very, very unfortunate focus. (P22)*

This participant seemed to acknowledge that working with suicidal patients might evoke a feeling of anxiety among therapists and that complying with procedures (risk assessment) may be perceived more as a type of personal protection. Thus, even though the risk assessment may be perceived as a necessary tool in the encounter with suicidal patients, it may also be a way for therapists to meet their own needs to protect themselves. Several participants described this need for such self-protection as unfortunate because it may obstruct a relational approach, which they perceived as fundamental to prevent suicide.

## General discussion

Through interviews with professionals implementing the national guidelines (and/or involved in relevant research), the purpose of the study was to explore how professionals working with suicide prevention experience the influence of the national guidelines on mental healthcare, and additionally, to gather recommendations for which steps to take next.

Although suicidality is commonly encountered in mental healthcare, our findings indicate that the national guidelines contributed to the field by drawing more attention to the topic of suicide prevention in mental healthcare. However, the participants believed that directives from the National Centre for Suicide Research and Prevention resulted in a constraining emphasis on standardized risk assessment during the implementation of the guidelines. Consequently, the professionals in this study had an ambivalent view on risk assessment—while it may be a useful tool to structure the encounter with suicidal patients, it may also compromise such important aspects as complexity, treatment and relations. Moreover, the participants criticized the Norwegian Directorate of Health Supervisions’ emphasis on risk assessment following suicides and suicide attempts in mental healthcare. The findings indicate that concerns over liability have become a burden for those working with suicidal patients, and that the need for self-protection affects professionals’ encounters with suicidal patients. Instead, the participants recommended a relational approach to suicide prevention where the main focus is on therapists establishing relationships with suicidal patients. According to Gergen (2009), we are relational beings from the outset. Relations are necessary if experiences such as hope, identity, meaningfulness and empowerment are to emerge (Price-Robertson et al., 2017). The participants’ call for a relational approach to suicide prevention in mental healthcare may therefore be understood on a more fundamental level as well. Hence, it is not enough to view the participants’ recommendation to establish a relationship to the patient as a call for a new procedure, but rather as a call for a fundamental change in our approach to and organization of suicide prevention. Price-Robertson et al. (2017) encourage us to develop, promote and implement approaches that “acknowledge the irreducibly relational nature of recovery” (p. 118).

The importance of and request for an emphasis on relations are well known (Østlie et al., 2018; Rasmussen & Dieserud, 2018). It is thus timely to ask *why* the national guidelines did not highlight relational aspects and why this still needs to be requested. Smith et al. (2015) claim that dependency on risk assessment is understandable for many reasons: if risk assessment had been possible it would have been a useful clinical tool; therefore, risk continues to be assessed. Moreover, risk assessment provides a clear goal, protects one from criticism and medio-legal action, structures communication and offers a sense of control. Furthermore, when working with something complex and difficult to treat, for example suicidality, it seems like we try to impose order onto chaos and therefore prefer a biomedical paradigm that is perceived to be more manageable (Brendel, 2006). As such, the emphasis on risk assessment may be an attempt to impose order and make suicidality more manageable. Here, we will point out two possible explanations for this on a more principled level.

First, we will look at the concept of knowledge. It is vital to understand the prevailing concept of knowledge because how we understand, describe and explain suicide influences national policies and prevention practices (Fitzpatrick et al., 2015; Marsh, 2016). According to Marsh (2016), the contemporary understanding of suicide is that it is pathological and individual in nature, and it is claimed that 90% of all suicides are associated with mental disorders (Cavanagh et al., 2003); hence, suicide prevention is the responsibility of specialist mental healthcare and psychiatry (Marsh, 2010; Walby et al., 2018). White et al. (2016) assert, for example, that:

“ *… the field of suicidology has become too narrowly focused on questions of individual pathology and deficit, as well as too wedded to positivist research methodologies, and thus has come to actively exclude from consideration approaches to understanding and preventing suicide that do not fit well with these orthodoxies*”. (p. 2)

The orthodoxy of individual pathology and deficit often emphasizes disease and diagnosis, and is thus described as objectifying (Borg et al., 2009; Marsh, 2010). Thus, a fundamental relational approach, where relations between people are understood as essential for recovery (Price-Robertson et al., 2017), may not fit well with the orthodoxy of individual pathology and deficit.

Moreover, the prevailing concept of knowledge involves an epistemological debate—what counts as true knowledge? Within suicidology, Fitzpatrick et al. (2015) describe this as a debate between the “objectivity” of nomothetic knowledge and more “subjective” idiographic approaches, where “objectivity” and thus quantitative methods prevail. Even though there are internal variations in disciplinary approaches to suicidality, the epistemological constructs are homogenous (Fitzpatrick et al., 2015). Marsh (2016) claims that an emphasis on categorizing, measuring and counting (nomothetic knowledge) makes it difficult to “understand and engage with the complex and changing context in which suicidal individuals are formed and suicides occur” (p. 28). Moreover, emphasis on an objective reality through scientific study obscures, disqualifies and marginalizes other kinds of knowledge (Marsh, 2010). The editor-in-chief of the most comprehensive suicide research journal, *Suicide and Life-Threatening Behaviour*, confirms Marsh’s (2010) assumptions when in an editorial he pronounces his preference for quantitative studies over qualitative studies (Joiner, 2011). Moreover, drawing on Foucault, Borg et al. (2009) claim that “true knowledge” is typically associated with having specialist knowledge, thus allowing professionals to have unique power and control” (p. 288). Hence, the medical discipline of psychiatry dominates the epistemology of mental health.

Both the service-user perspective and recovery literature challenge the orthodoxy of individual pathology and psychiatry dominating the epistemology of mental health (Borg et al., 2009; Webb, 2010). In contrast to an emphasis on individual pathology, the service-user perspective and recovery literature highlight, for example, relationships (Rasmussen & Dieserud, 2018; Topor et al., 2006), everyday life (Borg & Davidson, 2008) and being acknowledged as a human being (Berg, Rortveit & Aase, 2017) as important aspects for recovery. Therefore, several researchers call for an epistemological acknowledgement of the users’ subjectivity and experiences in both research and practice (Hjelmeland, 2016; Kogstad et al., 2011; Webb, 2010).

However, knowledge from service users and knowledge that highlights complexity and context are mostly acquired through qualitative research (Hjelmeland, 2016; Webb, 2010). Qualitative research is rarely implemented because “they are not on the golden standard list of evidence-based practice (EBP)” (Borg et al., 2009, p. 288). The national guidelines’ knowledge base builds on a literature review of suicide prevention measures within mental healthcare, mostly primary studies on the effect of psychotherapy, medication treatment and ECT, as well as systematic reviews of measures to improve continuity in and access to treatment (Walby, 2008). Hence, both the prevailing concept of knowledge and the epistemological debate may be part of the reason why the literature review (Mehlum et al., 2006, 2007) preceding the development of the national guidelines did not incorporate qualitative research highlighting relational approaches.

A second explanation can be viewed in light of the Norwegian hospital reform influenced by NPM’s ideologies. Even though the reform was an attempt to improve healthcare services by making them more equal and transparent, and thus leading to more accountable healthcare workers and more empowered patients (Byrkjeflot, 2011), the reform has also had some negative consequences. One consequence has been the expanding monitoring and reporting requirements (Lægreid et al., 2005; Wyller et al., 2013), which Wyller et al. (2013) claim signal a fundamental distrust in health workers. The emphasis on risk assessment can thus be an example of the expansion of monitoring and reporting, signalling a distrust in therapists’ clinical autonomy. Furthermore, Wyller et al. (2013) assert that another consequence of NPM is that healthcare workers find themselves in a moral conflict between loyalty to the “the line” and loyalty to the individual patient. When one of the participants said, “We don’t want the suicides, and we don’t want the blame”, this can be seen as an illustration of this moral conflict. Loyalty to “the line” means carrying out risk assessment as required and thus avoiding blame, whereas loyalty to the patient means to listen to the patient and establish a trusting relationship. The findings in this study indicate that loyalty to the patient requires some degree of clinical autonomy to be able to establish and maintain relationships. Accordingly, a critique of NPM is that it undermines professionals’ autonomy, where they may be less attentive to relational aspects in their encounters with patients (Wyller et al., 2013).

Moreover, the quest for transparency has been another part of NPM. Consequently, more reporting and extended rule systems are required, where the provision of guidelines has been one technique that has been applied (Blomgren & Sahlin, 2017). According to Blomgren and Sahlin (2017), economy, organization (politicians acting at a distance), awareness of patient rights, citizens’ interests in tax spending and transparency as a prerequisite for knowledge transfer are motivated by the quest for transparency. Nevertheless, Blomgren and Sahlin (2017) claim that a consequence of transparency can be that one tries to “avoid making mistakes rather than doing things right” (p. 176), a phenomenon described by Power (2004) as “secondary risk”. Moreover, an indirect consequence of transparency may be increased formalism and reporting (Blomgren & Sahlin, 2017). The quest for transparency and the following consequences may also explain the conflict of “we don’t want the blame and we don’t want the suicides”, which one of the participants highlighted. On the one hand, risk assessment may satisfy the quest of transparency because we can see what therapists have said and done. On the other hand, it seems that risk assessment may make professionals more preoccupied with avoiding mistakes and liability, and thus less free to do what they think is right, which is establishing relationships with the suicidal patients. Furthermore, as Blomgren and Sahlin (2017) assert:

It may well be that the quest for transparency, in its eagerness to find ways of making things visible, measuring, evaluating, and comparing, has come above all to stress whatever is convenient to make visible, whereas much of the important work that is actually performed in healthcare and has an impact on healthcare outcomes and development at least thus far has not been reducible to measurable and comparative categories. (p. 176)

Risk assessments can be made visible, they can be measured and evaluated, but the important work of establishing and maintaining relationships with suicidal patients cannot be reduced to measurable and comparative categories.

Consequently, the prevailing objectifying concept of knowledge, the epistemological debate and the New Public Management ideology may be impeding the use of a relational approach. This may be one of the reasons why the literature review (Mehlum et al., 2006, 2007) preceding the development of the national guidelines did not incorporate qualitative research, which has been calling for more relational approaches for a long time.

## Conclusion

The findings in this study challenge the current emphasis on standardization and risk-factor-based suicide risk assessment, and call for a fundamental change in suicide prevention towards an emphasis on relational aspects. Thus, it appears that we primarily need a paradigm shift when it comes to the concept of knowledge, which forms our understandings and, in turn, our practices.

## Strengths and limitations of the study

Although we conducted this study in a Norwegian context, the discussion regarding standardized suicide risk assessment and its consequences is relevant for an international audience as well. A limitation might have been the lack of participation from the National centre. However, this is counterbalanced through the diversity of participants with extensive experience from all other relevant institutions throughout Norway.

In this study, we have aimed at establishing trustworthiness through the criteria of credibility, dependability, confirmability and transferability (Nowell et al., 2017). We have addressed the first three criteria by describing how the study was conducted, by providing examples regarding the analysis process, and by describing how we created the themes through researcher triangulation and team consensus, respectively (Nowell et al., 2017). Even though the study was not conducted within a particular setting, it builds on the experiences and reflections from a diversity of participants within the suicide prevention community. Thus, we have aimed at providing thick descriptions regarding the background of the study and its context, as well as describing the participants included. Hence, the reader may evaluate the transferability to other settings (Polit & Beck, 2010).
